# Alterations of cerebellar white matter integrity and associations with cognitive impairments in schizophrenia

**DOI:** 10.3389/fpsyt.2022.993866

**Published:** 2022-09-26

**Authors:** Xuebin Chang, Xiaoyan Jia, Yulin Wang, Debo Dong

**Affiliations:** ^1^Department of Information Sciences, School of Mathematics and Statistics, Xi’an Jiaotong University, Xi’an, China; ^2^The Key Laboratory of Biomedical Information Engineering, Ministry of Education, Department of Biomedical Engineering, School of Life Science and Technology, Xi’an Jiaotong University, Xi’an, China; ^3^Key Laboratory of Cognition and Personality, Southwest University (SWU), Ministry of Education, Chongqing, China; ^4^Faculty of Psychology, Southwest University (SWU), Chongqing, China; ^5^Institute of Neuroscience and Medicine, Brain and Behaviour (INM-7), Research Centre Jülich, Jülich, Germany

**Keywords:** schizophrenia, cerebellum, cerebellar peduncle, white matter, cognitive impairment

## Abstract

“*Cognitive dysmetria” theory of* schizophrenia (SZ) has highlighted that the cerebellum plays a critical role in understanding the pathogenesis and cognitive impairment in SZ. Despite some studies have reported the structural disruption of the cerebellum in SZ using whole brain approach, specific focus on the *voxel*-*w*ise *changes of cerebellar WM microstructure and its associations with cognition impairments in SZ were less investigated.* To *further explore the voxel*-*w*ise *structural disruption of the cerebellum in SZ, the present study comprehensively examined* volume and diffusion features of cerebellar white matter in SZ at the voxel level (42 SZ vs. 52 controls) and correlated the observed alterations with the cognitive impairments measured by MATRICS Consensus Cognitive Battery. Combing voxel-*based morphometry (VBM) and diffusion tensor imaging* (DTI) method*s, we found, compared* to *healthy controls (HCs*), SZ patients did not show significant alteration in voxel-*level cerebellar white matter* (WM) volume and tract-*w*ise *and skele*to*nized* DTI features. In voxel-*w*ise *DTI features of cerebellar peduncles, compared* to *HCs, SZ patients showed decreased fractional anisotropy* and increased radial diffusivity mainly located in left middle cerebellar peduncles (MCP) *and inferior cerebellar peduncles* (ICP). *Interestingly, these* alterations were correlated with overall composite and different cognitive domain (including processing speed, working memory, and attention vigilance) in HCs but not in SZ patients. The present findings suggested that the voxel-*w*ise *WM integrity analysis might be a more sensitive way* to *investigate the cerebellar structural abnormalities in SZ patients. Correlation results suggested that inferior and* MCP *may be a crucial neurobiological substrate of cognition impairments in SZ*, thus *adding the evidence for taking the cerebellum as a novel therapeutic target for cognitive impairments in SZ patients.*

## Introduction

Schizophrenia (SZ) is a devastating disease with suspected neurodevelopmental origins and a life trajectory (1). Since SZ has been recognized as a brain disease, neuroscience has been attempted to unravel the neuropathological mechanism of SZ (2). In recent years, advances in magnetic resonance imaging (MRI), especially diffusion-weighted imaging (DWI) and high-resolution structural imaging (T1), have led to a new wave of research revealing white matter (WM) connectivity interruptions in patients with SZ. Most of the existing work has used well-established and widely used diffusion metrics, such as fractional anisotropy (FA), mean diffusivity (MD), axial diffusivity (AD), and radial diffusivity (RD) to characterize the microstructure of global WM in SZ (3) with a particular interest in cerebral WM tracts (4), and mainly found changes in the frontotemporal, interhemispheric, and frontal thalamic WM tracts (5, 6). Reductions in FA are considered to be a sign of myelin abnormalities and/or axonal impairment (7). However, there is a lack of specific focus on the cerebellar WM microstructure in SZ in the literature.

Traditionally, the cerebellum is thought to be mainly dedicated to motor coordination (8). However, in recent years, numerous studies suggested that the cerebellum not only contributes to control of action but also involves in high-level cognitive and emotional functions (9–13). Last two decades, the critical role of the cerebellum in the pathogenesis and cognitive impairments of SZ has been emphasized by the “cognitive dysmetria” theory (also referred to as the “dysmetria of thought” theory). And previous animal and human neuroimaging studies have provided converging evidence for the involvement of cerebellar function in various behaviors that are dependent on circuits connecting the cerebellum with multiple cerebral cortical regions (14).

The output fibers of the cerebellum (excluding the vestibular cerebellum to the vestibular nucleus) primarily originate from the four deep cerebellar nuclei: the dentate nucleus, the embolic nucleus, the globular nucleus, and the parietal nucleus. The superior cerebellar peduncle (SCP) is the mainly cerebellar efferent pathway that connects the cerebellum to cerebral regions through the thalamus. In addition, the inferior cerebellar peduncles (ICP) contain efferent connections from the cerebellum to the vestibular nuclei (15, 16). All input fibers of the cerebellum need to pass through the middle cerebellar peduncles (MCP) (15). After the cerebellar structural and functional lesion, patients with neurological disorders were found to exhibit a range of cognitive deficits, including impaired executive function, spatial cognition, language processing, and emotional regulation (17). Cerebellar dysfunction has been proposed to explain the cognitive-affective deficits and symptom heterogeneity observed in SZ (13). Consistent with this idea, existing studies have reported that patients with SZ have reduced volume in the cerebellar vermis (18). In addition, the SZ patients showed the disrupted network topography architecture of cerebellum in SZ (9, 19, 20). Some studies investigated the structural WM disruption of the cerebellum in SZ often using parcellation-based approach (21–23). Using whole brain voxel-wise approach, some studies have reported cerebellar and cerebral WM abnormalities in first episode SZ (24, 25). To the best of our knowledge, only one study investigated the voxel-wise abnormalities of cerebellar WM skeletonized features using Tract-Based Spatial Statistics (TBSS) and evaluated its associations with cognition function in SZ (26). This study found decreased FA in the MCP in SZ and such alteration was associated with cognitive impairments in SZ. Given that this study was mainly focused on the deep WM of cerebellum, more studies are needed to explore and validate the findings of this study and further investigate the voxel-wise WM abnormalities of cerebellum not only in deep WM but also in all regions of cerebellar WM peduncles (27).

The purpose of this study is to comprehensively examine volume and diffusion features of cerebellar WM in SZ at voxel level (42 SZ vs. 52 controls) and correlate the observed alterations with the cognitive impairments measured by Measurement and Treatment Research to Improve Cognition in SZ (MATRICS) Consensus Cognitive Battery. Specifically, Cerebellar-specific voxel-based morphometry (VBM) analysis was performed using the Spatially Unbiased Infratentorial template to characterize cerebellar WM volume. Diffusion metrics (FA, MD, AD, and RD) of cerebellar WM were calculated from the diffusion tensor imaging (DTI) data. We hypothesized that SZ patients would show altered WM features, and such alteration would correlate with the cognitive deficits in SZ patients.

## Materials and methods

### Participants

This study included 42 SZ patients and 52 healthy controls (HCs). The imaging and phenotypic information of data were downloaded from the Collaborative Informatics and Neuroimaging Suite Data Exchange tool (COINS)^1^ (28) and data collection was performed at the Mind Research Network, funded by a Center of Biomedical Research Excellence (COBRE) grant from the National Institutes of Health. The diagnostic confirmation of SZ was confirmed by the Structured Clinical Interview for DSM-IV Axis I Disorders. Psychopathological symptoms of SZ were evaluated using the Positive and Negative Syndrome Scale (PANSS) (29). All patients were treated with antipsychotics, and the antipsychotic medication was converted to chlorpromazine equivalents. The MATRICS Consensus Cognitive Battery (MCCB) cognitive battery of all participants was additionally included in this study. All participants were excluded for a history of substance abuse or dependence within the last 12 months, a history of neurological illness, and traumatic brain injury. Written informed consent was obtained from all participants according to institutional guidelines required by the Institutional Review Board at the University of New Mexico (UNM). Five patients and three HCs were excluded because the whole cerebellum was not fully covered during the scanning of the T1 and/or DTI. Finally, 37 SZ patients and 49 HCs were included in the final analysis. The detailed demographic, clinical, and cognitive information of all patients and HCs are shown in Table 1.

**TABLE 1:** Demographic characteristics of the schizophrenia patients and healthy controls.

Variables	SZ (*n* = 37)		HC (*n* = 49)		*P*-value
	Mean	*SD*	Mean	*SD*	
Age (years)	38.73	13.79	38.90	12.07	0.952
Gender (male: female)	28: 9		36: 13		0.816
Handedness (right: left: both)	34: 2: 1		45: 2: 2		0.907
Processing speed	34.51	11.59	53.73	8.14	< 0.001
Attention vigilance	33.86	13.73	50.36	9.91	< 0.001
Verbal working memory	37.46	13.70	48.22	11.08	< 0.001
Verbal learning	37.86	8.36	45.02	6.59	< 0.001
Visual learning	35.43	11.64	46.84	9.85	< 0.001
Reasoning problem solving	42.00	10.25	54.70	7.66	< 0.001
Social cognition	40.35	11.97	52.78	9.78	< 0.001
Overall composite	29.25	12.83	49.74	8.98	< 0.001
Chlorpromazine equivalents (mg/d)	396.78	354.14	–	–	
Duration of illness (years)	18.19	13.77	–	–	
PANSS-positive	14.35	4.60	–	–	
PANSS-negative	15.03	5.45	–	–	
PANSS-general	29.35	8.07	–	–	
PANSS-total	58.73	13.71	–	–	

SZ, schizophrenia; HC, healthy controls; SD, standard deviation; PANSS, Positive and negative Syndrome Scale.

### Data acquisition

All images were collected on a 3-T Siemens Trio scanner with a 12-channel radio-frequency coil at the Mind Research Network. High resolution T1-weighted structural images were obtained using a five-echo MPRAGE sequence with following imaging parameters: time of repetition (TR) = 2.53 s, echo time (TE) = 1.64, 3.5, 5.36, 7.22, 9.08 ms, inversion time (TI) = 1.2 s, flip angle = 7°, filed of view (FOV) = 256 × 256 mm, number of excitations = 1, slice thickness = 1 mm. The scan parameters of DTI were as follows: TR = 9 s; TE = 84 ms; field of view (FOV) = 256 × 256 mm; slice thickness = 2 mm; number of slices = 72; slice gap = 2 mm; voxel resolution 2 × 2 × 2 mm; flip angle = 90°; number of diffusion gradient directions = 35, *b* = 800 s/mm^2^. All images of DTI were registered to the first *b* = 0 image.

### Cognitive testing

To evaluate cognitive ability, the test of MATRICS Consensus Cognitive Battery was conducted for each participant (30). MATRICS measures cognitive performance in seven domains: processing speed, attention/vigilance, verbal working memory, verbal learning, visual learning, reasoning, problem solving, and social cognition. MATRICS has been regarded as the standard tool for comprehensively assessing cognitive deficits in individuals diagnosed with SZ and related disorders with excellent reliability and validity (30).

### Voxel-based morphometry analysis

To investigate the structural morphological characteristics of cerebellar WM in patients with SZ, the cerebellar-specific VBM analysis was performed using the Spatially Unbiased Infratentorial template (SUIT)^2^ (31) toolbox implemented in Statistical Parametric Mapping, Version 12 (SPM 12).^3^ Before the calculation of VBM, quality control of T1 images was carried out, and subjects without a complete cerebellar scan were excluded in the subsequent analysis. The steps of VBM analysis were as following (32). First, individual T1-weighted sequences were manually reoriented the image origin at the anterior commissure. Next, the segment and isolate the function of SUIT were used to isolate the infratentorial structure (cerebellum and stem) from the surrounding tissue and segment the infratentorial structure into WM, gray matter, and cerebrospinal fluid. Then, the individual WM was normalized to the SUIT space using the Diffeomorphic Anatomical Registration Through Exponentiated Lie Algebra (DARTEL) algorithm and modulated by the deformation fields to preserve the original volume of the tissue. Finally, the resulted WM volume maps were smoothed using a 6 mm full width at half-maximum (FWHM).

### Diffusion tensor imaging analysis

To investigate the structural diffusion features of cerebellar WM in patients with SZ, the DTI data were analyzed using the FMRIB Software Library (FSL).^4^ First, non-brain tissues were removed from the DTI data using the brain extraction tool algorithm in FSL. Next, head motion and eddy current corrections were carried out by the affine transformation between the gradient images and the baseline *b* = 0 image. Then, diffusion tensors were calculated using drift tool in FSL, and subsequently, FA, MD, AD, and RD maps were obtained. Besides, all subjects’ FA maps were aligned with the Montreal Neuroimaging Institute (MNI 152) template space using the non-linear registration tool FNIRT. Furthermore, the deformation fields from FA maps were used to project the registered MD, AD, and RD maps onto the FA skeleton. Finally, the resulted maps were smoothed using a 6 mm FWHM.

### Statistical analysis

The independent *t*-tests and chi-square tests were used to compare the continuous and categorical variables of demographic characteristics separately between patients and HCs.

The significant group difference in VBM between patients and HCs was determined by permutation-based non-parametric test with 5,000 permutations and using the threshold-free cluster enhancement (TFCE) method in FSL Randomize (33), and age, gender, and cerebellar WM volume were regressed out as covariates. The significance was set at *p* < 0.05, family wise error (FWE) corrected for multiple comparisons.

Voxel-wise comparison of DTI features within the three cerebellar peduncles (27) between patients and HCs was performed using the same statistical method of volume analysis. Results with a cluster extent threshold of 100 contiguous voxels were reported. The statistical maps of the analyses were binarized at the threshold of *p* < 0.05, FWE corrected for multiple comparisons. Then, the binarized maps were multiplied to create cerebellar WM masks to determine WM changes within the cerebellum. Besides, between-group voxel-wise comparisons of cerebellar skeleton were conducted using TBSS.^5^ The cerebellar skeleton obtained by multiplying the mean FA skeleton mask by the regional mask of cerebellar peduncles (27). The voxel-wise comparisons of DTI features within cerebellar skeleton were performed using permutation-based non-parametric testing with 5,000 permutations, with age, gender, and cerebellar WM volume included as nuisance covariates. The statistical significance was set at *p* < 0.05 after adjusting for multiple comparisons using the TFCE method in FSL Randomize (33).

In terms of statistical analysis of tract-wise DTI features, we used the probabilistic atlas of cerebellar WM in the MNI152 space and created masks of three pairs of cerebellar peduncles (27). The FA map was then multiplied to create inclusive masks with the masks of cerebellar peduncles. The average FA values from each tract were extracted by averaging all voxels belonging to the tract. The between-group comparisons of tract-wise FA values of each tract were analyzed using the Mann-Whitney test with age, gender, and cerebellar WM volume included as nuisance covariates. In addition, similar processing and statistics were also carried out in MD, AD, and RD maps. The statistical significance was set at *p* < 0.05 (false discovery rate corrected).

Finally, to investigate the correlation between altered WM features of the cerebellum and the cognition assessments in the patient group and the HCs group, respectively, we calculated the Spearman correlations between the overall composite assessment and altered WM features within each group since the data of DTI metrics were not normally distributed (Shapiro-Wilk *W*-test, *p* < 0.05). Meanwhile, to help clarify the specific correlation between different cognitive domain and altered WM features, we also conducted correlation analyses between each cognitive domain and altered WM features as exploratory analysis without controlling the multiple testing correction.

## Results

### Cognitive performance

As expected, SZ patients showed cognitive deficits across all the seven domains: processing speed, attention/vigilance, verbal working memory, verbal learning, visual learning, reasoning, problem solving, and social cognition (Table 1). The group of SZ patients matched well with the group of healthy controls at basic demographic variables, i.e., age, gender, and handedness.

### Voxel-based morphometry analysis

To investigate the structural morphological differences in cerebellar WM between SZ patients and HCs, we contrasted the cerebellar WM volume maps between the two groups. The SZ patients did not differ from HCs regarding the cerebellar WM volume at voxel level.

### Diffusion tensor imaging analysis

**FIGURE 1:**
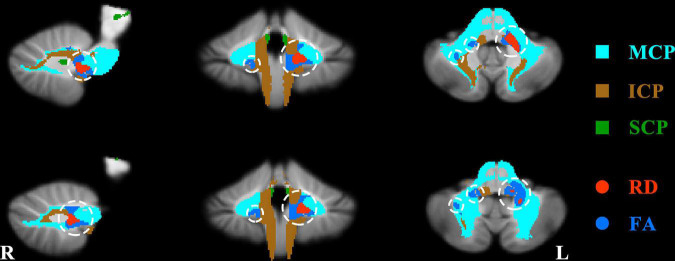
Significant group difference about fractional anisotropy (FA) and radial diffusivity (RD) between patients and healthy controls. The regions of significant increased RD and decreased FA in the patients were shown with red and dark blue separately and circled with white circles. MCP, middle cerebellar peduncles; ICP, inferior cerebellar peduncles; SCP, superior cerebellar peduncles.

In voxel-wise DTI features, compared to HCs, SZ patients showed WM changes in a region across MCP and ICP. In detail, SZ patients showed decreased FA in left ICP and right MCP (Figure 1 and Table 2) and increased RD in left MCP (Figure 1 and Table 2). The significant group differences were mainly located in the left cerebellum (Figure 1). The SZ patients did not differ from HCs regarding MD and AD. Besides, no significant group difference was found in terms of cerebellar skeletonized DTI metrics.

**TABLE 2:** Significant differences of voxel-wise DTI metrics between SZ and HC.

DTI metrics	Brain regions	MNI coordinates			Cluster size	Peak *p*-value
		*x*	*y*	*z*		
FA (SZ < HC)	ICP	−10	−39	−45	1,729	0.002
	MCP	18	−44	−41	248	0.016
	MCP	29	−52	−36	199	0.041
RD (SZ > HC)	MCP	−18	−49	−40	480	0.005

SZ, schizophrenia; HC, healthy controls; DTI, diffusion tensor imaging; MNI, Montreal Neurological Institute; FA, fractional anisotropy; RD, radial diffusivity; MCP, middle cerebellar peduncles; ICP, inferior cerebellar peduncles.

In tract-wise DTI features, no significant difference was found between SZ patients and HCs in any DTI features.

### Correlations between altered white matter features and cognitive assessments

**FIGURE 2:**
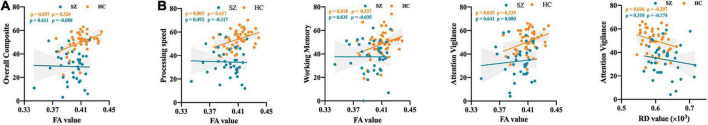
The correlation between altered diffusion features and cognitive assessments. **(A)** The correlation between altered diffusion features and overall composite. **(B)** The correlation between altered diffusion features and different cognitive domain. SZ, schizophrenia; HC, healthy controls; FA, fractional anisotropy; RD, radial diffusivity.

For the correlations between altered WM features and overall composite assessment, a significant positive correlation was found between the mean FA value in the altered region across ICP and MCP and overall composite in HCs but not in SZ patients. The mean FA value in the altered region in HCs was positively correlated with overall composite (ρ = 0.320, *p* = 0.037, Figure 2A), but no significant correlation was found in SZ patients (Figure 2A).

Besides, for the correlations between altered WM features and different cognitive domain, a significant positive correlation was found between mean FA value in the altered region and different cognitive domain in HCs but not in SZ patients. Similarly, a significant negative correlation was observed between the mean RD value in the altered region and different cognitive domain in HCs but not in SZ patients. In detail, the mean FA value in the altered region in HCs was positively correlated with processing speed (ρ = 0.417, *p* = 0.003), working memory (ρ = 0.337, *p* = 0.018), and attention vigilance (ρ = 0.335, *p* = 0.025), but no significant correlation was found in SZ patients (Figure 2B). Besides, the mean RD value in the altered region in HCs was negatively correlated with attention vigilance (ρ = -0.296, *p* = 0.046), but no significant correlation was found in SZ patients (Figure 2B).

Furthermore, we also investigated the Spearman correlation between cognitive assessments and mean FA values in three cerebellar peduncles (27) separately as exploratory analysis. Similar to the main findings, cognitive assessments correlated with mean FA values in cerebellar peduncles in HCs but not in SZ patients (Supplementary Figure 1). Besides, to be reassuring that the findings observed in ENIGMA consortium (34) can be replicated in the COBRE dataset, we evaluated the group difference of mean FA in anterior corona radiata (the most associated with cognition in ENIGMA study) and further investigated the Spearman correlation between cognitive assessment and mean FA in anterior corona radiata. Compared with HCs, SZ patients showed decreased FA in anterior corona radiata (*t* = -3.29, *p* = 0.002). The mean FA values in anterior corona radiata in HCs was positively correlated with attention vigilance (ρ = 0.302, *p* = 0.044), but no significant correlation was found in SZ patients.

## Discussion

To the best of our knowledge, this is the first study to comprehensively investigate the WM features of the cerebellum at the voxel-level in patients with SZ, and further assess the correlation between altered WM features and cognitive assessments in SZ. The key findings of this study were that we observed voxel-wise WM abnormalities (FA and RD) mainly across the left MCP and ICP. However, no significant difference was found between SZ patients and HCs in any tract-wise and skeletonized DTI features and voxel-level cerebellar WM volume. Importantly, significant correlations between the altered WM features and cognitive assessments only revealed in HCs but not in SZ patients. The present findings suggested that the voxel-wise WM integrity analysis might be a more sensitive way to investigate the cerebellar WM abnormalities in SZ patients. And these findings also highlighted the important role left MCP and ICP in cognitive disruption in SZ.

Previous studies have investigated the WM structural connectivity (35–37) or VBM (38–40) in the whole brain in SZ patients. Although a previous meta-analysis study has investigated changes of gray matter in the cerebellum (41), no study has comprehensively focused on cerebellar WM abnormalities by a combined VBM and DTI method. This study filled this gap and found that SZ patients did not show significant abnormality in cerebellar WM volumes and significant abnormality in tract-wise and skeletonized WM structural connectivity while showing decreased FA and increased RD mainly in a region across left MCP and ICP in voxel-wise WM structural connectivity. These findings were consistent with the previous study that evidenced the voxel-based diffusion data analysis is more sensitive than tract-wise analysis in identifying WM abnormalities (36). Besides, despite the analysis of voxel-wise cerebellar WM structural connectivity revealed significant effect in cerebellar peduncles but not cerebellar skeleton in our work. This findings was inconsistent with Kim et al.’s study (26), which demonstrated significant effect in the cerebellar skeleton. Interestingly, we found significant decreased FA in MCP, which was consistent with the impaired regions observed in Kim et al.’s study (26). These points highlighted future studies with large sample size are needed to further validate these observed results. Previous study indicated that reduction of FA might reflect damage or disordered WM and fiber structure caused by axonal loss or demyelization while elevation of RD can result from reduced myelin integrity (7). Therefore, we suspected that decreased FA together with increased RD might reflect demyelination of the cerebellum in patients with SZ. Interestingly, our previous meta-analysis study documented that, compared to HCs, SZ patients exhibited widespread reduced FA in the left side of the brain (6), and the previous WM studies of whole brain also found that such changes were mainly located in the left side of the brain in SZ (42, 43). The present observed that such changes in WM of cerebellum were located in the left cerebellum, which provided further evidence for the leftward changes in some key white-matter tracts in SZ (44). It should be noted that the cerebellar MCP and ICP peduncles, as the input fiber of the cerebellum, are the main pathway to communicate with the cerebrum and cerebellum. Decreased FA and increased RD in cerebellar peduncles in SZ patients might be related to the cerebro-cerebellar dysconnectivity (26, 45). In addition, in VBM, we did not find significant abnormality in cerebellar WM volume in SZ patients. In SZ, although FA changes are usually associated with atrophy, they may not have volume changes depending on the method, the region studied and the underlying pathological changes (46). Collectively, the present study provided precise location for the changes of cerebellar WM in SZ and observed changes of WM integrity in MCP and ICP provided a further structural basis for the well-documented abnormal cerebellar-cerebral functional connectivity in SZ (9, 47, 48).

Interestingly, the cognitive assessments were positively correlated with FA and negatively correlated with RD in left cerebellar peduncles in HCs but not in SZ patients. Similarly, the cognitive assessments were positively correlated with FA in anterior corona radiata in HCs but not in SZ patients. These findings were conceptually similar to the previous study that demonstrated the positive correlation between FA in inferior and middle frontal gyrus and cognitive assessments in HCs but not in patients with SZ (43). This finding not only suggests that the ACR alteration can be replicated in the present study but also implies that prior large-scale studies such as ENIGMA may have missed a significant finding in cerebellar peduncle by excluding the cerebellum from comparisons of WM differences between schizophrenia and controls. In addition, we observed significant positive correlation the mean FA values of anterior corona radiata and cognition function in HCs but not in SZ groups. This finding was not consistent with Kochunov et al.s’ study, which observed such correlation both in SZ patients and HCs. Such inconsistence calls on future studies to pay more attention on the heterogeneity of the included sample. Besides, previous studies demonstrated that executive dysfunction is one of the most common dysfunctions in the course of SZ (49, 50), the observed impairments across all the domains of MATRICS further supported this idea. The integrity of the cerebellar peduncles WM connectivity plays a crucial role in the reciprocal communication between the cerebellum and the cerebral cortex (10), thus it can reasonably explain that the FA of the cerebellar peduncles will be related to the processing speed and attention vigilance in HCs but not in SZ patients. Functional imaging studies have suggested that the dysfunction of the prefrontal cortex is a critical neural substrate for cognitive dysfunction in SZ *via* hypoconnectivity with prefrontal-cerebellar regions (especially during working memory tasks) (51–53). Our results showed that cerebellar peduncles predicted attention and working memory behavioral performance in healthy subjects, supporting the fact that cerebellar MCP and ICP have a critical role in working memory and attention performance in healthy controls (54, 55). However, the cerebellar WM–cognition relationships were disrupted in patients with SZ. This result suggests that cerebellar peduncles, i.e., MCP and ICP, might be a meaningful neurobiological basis for cognitive performance and a novel therapeutic target for cognitive impairment in SZ patients.

Notwithstanding its implications, the limitations of this study should be acknowledged. The relatively small samples of patients and controls were enrolled in this study, which might limit the generalization of the observed findings. Nonetheless, the current study still provides some evidence supporting that the WM of the cerebellum plays a critical role in the cognitive impairments of SZ. The other limitation is the effect of antipsychotic drugs, a common issue in many other studies in the field. While we cannot eliminate the effects of medication on WM structures and cognition impairments, we found that the altered WM of the cerebellum still did not correlate with cognitive assessments in SZ group after regressed out the Chlorpromazine equivalents (*p* > 0.05), suggesting that these associations are unlikely to be mainly driven by medication. Besides, the psychiatric comorbidities are common issue of patients with SZ, which might affect the observed results. However, the dataset of COBRE did not provide the information of comorbidities, which limit us to evaluate the potential effect of the comorbidity on the observed results.

In summary, we found voxel-wise WM abnormalities (FA and RD) in the left MCP and ICP of the cerebellum. We did not find tract-wise and skeletonized WM structural connectivity and volume abnormality of the cerebellum in patients with SZ. These results might suggest that the voxel-wise WM diffusion data analysis is more sensitive than tract-wise analysis in identifying WM abnormalities of cerebellum in SZ patients. Our correlation analyses showed that the FA of MCP and ICP was significantly associated with processing speed in HCs but not in SZ patients, suggesting that cerebellar peduncles might be a meaningful neurobiological basis of cognitive impairments and a novel therapeutic target for cognitive impairments in SZ patients.

## Data availability statement

The raw data supporting the conclusions of this article will be made available by the authors, without undue reservation.

## Ethics statement

The studies involving human participants were reviewed and approved by the University of New Mexico. The patients/participants provided their written informed consent to participate in this study.

## Author contributions

XC, XJ, and DD generated the idea of the study. XC and XJ downloaded the data and finished the calculation. XC, XJ, YW, and DD drafted and revised the manuscript. All authors contributed to the article and approved the submitted version.

## Footnotes

1
http://coins.mrn.org/dx

2
http://www.diedrichsenlab.org/imaging/suit.htm

3
https://www.fil.ion.ucl.ac.uk/spm/software/spm12/

4
www.fmrib.ox.ac.uk/fsl

5
https://fsl.fmrib.ox.ac.uk/fsl/fslwiki/TBSS

## Supplementary material

Supplementary Figure 1 The correlation between mean fractional anisotropy in cerebellar peduncles and cognitive assessments. **(A)** The correlation between mean fractional anisotropy in cerebellar peduncles and overall composite. **(B)** The correlation between mean fractional anisotropy in cerebellar peduncles and different cognitive domain. SZ, schizophrenia; HC, healthy controls; FA, fractional anisotropy; ICP, inferior cerebellar peduncles; MCP, middle cerebellar peduncles; SCP, superior cerebellar peduncles.
