# Supplementary figures and images for: Alterations of cerebellar white matter integrity and associations with cognitive impairments in schizophrenia
Source: Front Psychiatry. 2022 Sep 26;13:993866. doi: 10.3389/fpsyt.2022.993866 (PMC9549145; doi:10.3389/fpsyt.2022.993866)

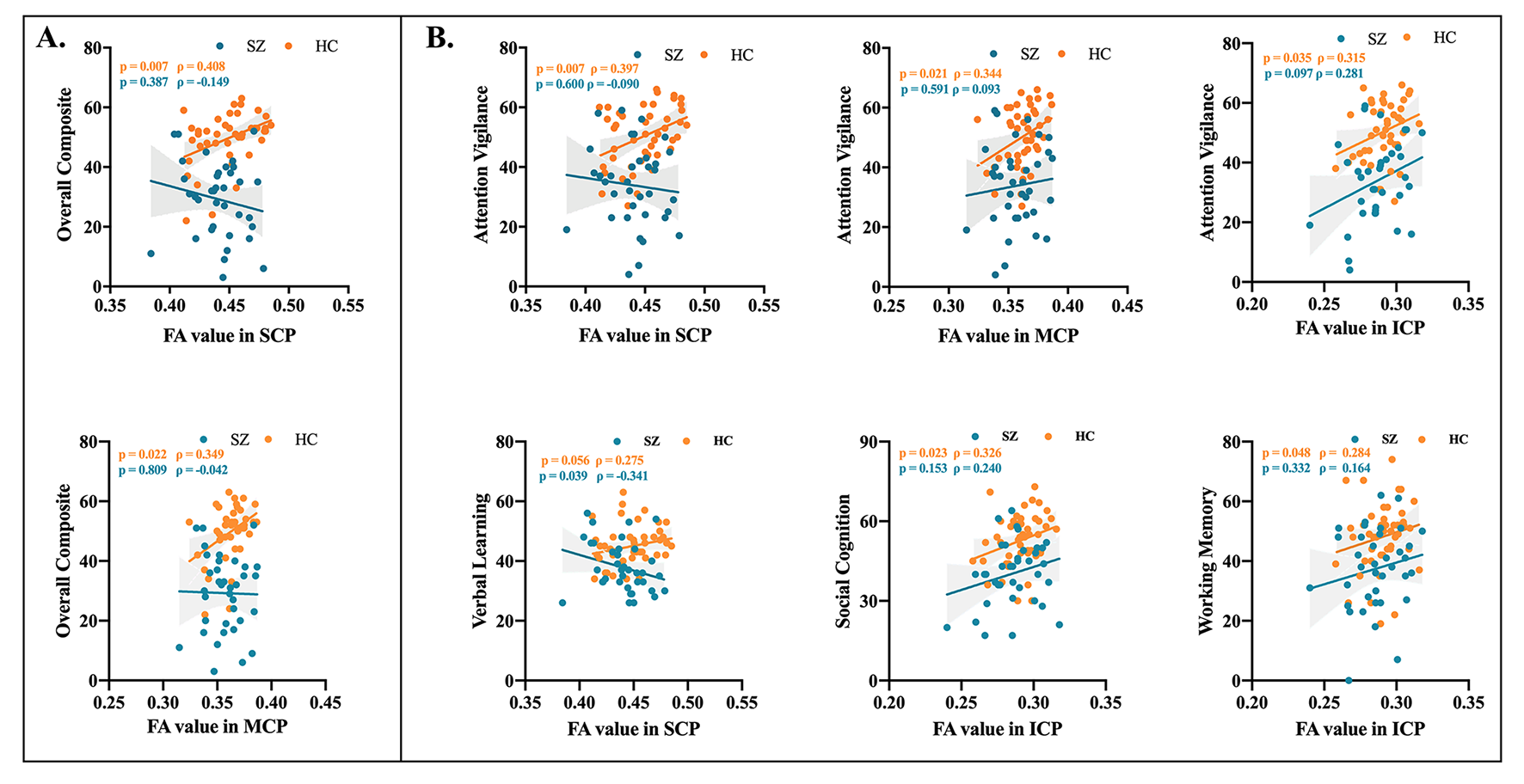

Supplement: Supplementary Figure 1 — The correlation between mean fractional anisotropy in cerebellar peduncles and cognitive assessments. (A) The correlation between mean fractional anisotropy in cerebellar peduncles and overall composite. (B) The correlation between mean fractional anisotropy in cerebellar peduncles and different cognitive domain. SZ, schizophrenia; HC, healthy controls; FA, fractional anisotropy; ICP, inferior cerebellar peduncles; MCP, middle cerebellar peduncles; SCP, superior cerebellar peduncles. [file Image_1.TIF]
